# *TARS2* c.470 C > G is a chinese-specific founder mutation in three unrelated families with mitochondrial encephalomyopathy

**DOI:** 10.1186/s13023-024-03365-w

**Published:** 2024-10-11

**Authors:** Shujie Zhang, Haisong Qin, Qingming Wang, Yingfei Wang, Yanhui Liu, Qi Yang, Jingsi Luo, Zailong Qin, Xiang Ji, Lijuan Kan, Guoxing Geng, Jing Huang, Shengkai Wei, Qiuli Chen, Yiping Shen, Haiming Yuan, Baoling Lai

**Affiliations:** 1 Department of Genetics and Metabolism, Maternal and Child Health Hospital of Guangxi Zhuang Autonomous Region, Nanning, 530003 P.R. China; 2 Department of Medical Genetics, Dongguan Maternal and Child Health Care Hospital, Dongguan, 523120 China; 3 Department of Medical Laboratory, Shenzhen Luohu People’s Hospital, the Third Affiliated Hospital of Shenzhen University, Shenzhen, 518000 P.R. China; 4 Shenzhen Luohu People’s Hospital, the Third Affiliated Hospital of Shenzhen University, Shenzhen, 518000 P.R. China; 5 Division of Genetics and Genomics, Boston Children’s Hospital, Harvard Medical School, 02115 Boston, MA USA

**Keywords:** *TARS2*, COXPD21, Chinese-specific, Founder mutation, Mitochondrial encephalomyopathy

## Abstract

Biallelic pathogenic variants in *TARS2* lead to combined oxidative phosphorylation deficiency, subtype 21 (COXPD21, MIM #615918), which is a rare mitochondrial encephalomyopathy (ME) characterized by early-onset severe axial hypotonia, limb hypertonia, psychomotor developmental delay, epilepsy and brain anomalies. To date, approximately 28 individuals with COXPD21 and 28 *TARS2* variants have been identified. In this study, we reported additional four individuals from three unrelated Chinese families with mitochondrial encephalomyopathy caused by pathogenic variants in *TARS2*, and described the novel clinical phenotypes and genotypic information. In addition to two novel variants (c.512G > A, p.Arg171Lys; c.988dup, p.Arg330Lysfs*4), one previously reported variant (c.470 C > G, p.Thr157Arg) recurred in six Chinese individuals with COXPD21 but was not present in populations of other races. Our findings expanded the mutation spectrum of *TARS2* and confirmed that c.470 C > G is a Chinese-specific founder mutation. The novel phenotypes, including reduced fetal movement, eye anomalies and sleep irregularities, observed in our patients enriched the clinical characteristics of COXPD21.

**Supplementary Information:**

The online version contains supplementary material available at 10.1186/s13023-024-03365-w.

## Introduction

Biallelic pathogenic variants in *TARS2* cause combined oxidative phosphorylation deficiency 21 (COXPD21), which is a rare mitochondrial encephalomyopathy characterized by early-onset severe axial hypotonia, limb hypertonia, delayed psychomotor development, epilepsy, brain anomalies and increased serum lactate level, often leading to early death; onset after 6 months results in a milder course and longer survival [1]. *TARS2*, a mitochondrial aminoacyl-tRNA synthetase (mt-aaRS), generates mitochondrial Thr-tRNA^Thr^ by catalyzing the binding of homologous tRNA to specific amino acids and clearing mischarged Ser-tRNA^Thr^ during mitochondrial translation, which safeguards normal and accurate mitochondrial protein synthesis. To date, 19 mt-aaRS genes have been identified to be involved in human mitochondrial disorders [2, 3]. Pathogenic variants in mt-aaRS genes lead to a reduction in charged tRNA, which impairs mitochondrial protein synthesis and decreases the amount of mtDNA-encoded subunits of the oxidative phosphorylation system (OXPHOS), ultimately resulting in combined respiratory chain enzyme deficiency [4–7]. *TARS2* (MIM 612805) encodes a 718-amino acid mitochondrial threonyl tRNA-synthetase, which contains an N-terminal N1 domain (Leu20-Ser124), an N2 domain (for editing) (Pro125-Asp301), an aminoacylation domain (for amino acid activation and tRNA charging) (His302-Gly605), and an anticodon binding domain (for tRNA binding) (Lys606-Phe718) [8]. To date, only 28 COXPD21 patients and 28 pathogenic variants in *TARS2* have been reported; thus, the genotype-phenotype relationships are not well established [1, 9–13]. Here, we present four COXPD21 patients from three unrelated Chinese families. Two novel *TARS2* variants were identified, and novel phenotypes were observed; these findings expand the mutation spectrum of *TARS2* and enrich the clinical characteristics of this disease. Furthermore, we identified *TARS2* c.470 C > G as a Chinese-specific founder mutation.

## Materials and methods

### Ethical compliance

This study was approved by the Ethics Committee of Maternal and Child Health Hospital of Guangxi Zhuang Autonomous Region, Dongguan Maternal and Child Health Care Hospital, and Shenzhen Luohu People’s Hospital. Written informed consent was obtained from the legal guardians for the publication of any potentially identifiable images or data included in this study.

### Whole-exome sequencing (WES)

Whole-exome sequencing was employed for our patients and their parents. Genomic DNA was extracted from peripheral blood samples using QIAamp DNA Blood Mini Kit (Qiagen, Germany). Library preparation was operated using the Agilent SureSelect Human All Exon kit V5 (Agilent, Santa Clara, CA). Bcl2fastq tool (v2.15.0.4) was applied for extracting Fastq files from Illumina bcl sequencing file. BWA (0.7.10-r789), Picard (v1.128) and Genome Analysis Toolkit (GATK v3.5) were performed for genome alignments and variant detection. The Annovar tool was used for variant annotation. Suspected variants were confirmed by Sanger sequencing. The pathogenicity of the sequence variants was assessed according to ACMG/AMP guidelines [14].

## Results

### Clinical phenotypes

#### Family 1

**Fig. 1:**
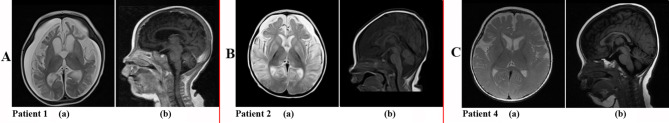
Brain MRI for our patients. Patient 1 (1 **A**): MRI showed bilateral underdeveloped insula, frontal, temporal and occipital lobes, microgyria, decreased white-matter volume, dilated bilateral lateral ventricles, bilateral symmetrical T2-weighted imaging (T2WI) multiple hyperintense lesions in the basal ganglia, thalamus and lenticular nucleus (**a**) and a thin corpus callosum (**b**); Patient 2 (1**B**): MRI showed frontal subdural fluid, bilateral hemispheric brain atrophy, multiple hyperintense lesions in the basal ganglia, globus pallidus, thalamus, midbrain and cerebral peduncles (**a**), and a thin corpus callosum (**b**); Patient 4 (1 **C**): Brain MRI revealed hyperintensity of the bilateral basal ganglia on symmetrical T2-weighted imaging (T2WI) (**a**) and a thin corpus callosum (**b**)

Patient 1 was an 8-month-old boy who was born to a healthy gravida 3, para 2 mother with a negative family history of genetic diseases. He was the first baby of this family, and his delivery at 40 weeks of gestation was uneventful except for obviously reduced fetal movement. He had normal birth measurements: weight 2.8 kg, length 50 cm and head circumference 33 cm. At 3.5 months of age, he exhibited axial hypotonia and could not raise his head. He displayed frequent blinking and nystagmus. Serum lactate level (2.69 mmol/L, normal range 0.5–1.7 mmol/L) was elevated. At 5 months of age, he suffered from intractable epileptic episodes characterized by staring, muscle rigidity, rapid blinking and autonomic symptoms. Electroencephalography (EEG) revealed rhythm weakness on the right side, with diffuse slow waves and epileptic discharges, particularly posteriorly. At 7 months of age, the patient had normal growth development: his height was 69 cm, and his weight was 9 kg. However, he had obvious microcephaly, and his head circumference was 39.5 cm (<-3 SD). Limb hypertonia manifested. Echocardiography revealed an atrial septal defect, diffuse hyperthermic cardiomyopathy and pericardial effusion. MRI showed bilateral underdeveloped insula, frontal, temporal and occipital lobes, microgyria, decreased white-matter volume, dilated bilateral lateral ventricles, bilateral symmetrical T2-weighted imaging (T2WI) multiple hyperintense lesions in the basal ganglia, thalamus and lenticular nucleus and a thin corpus callosum (Fig. 1A). His serum lactate level was increased (8.79 mmol/L). The patient passed away at 8 months of age due to respiratory failure.

**Fig. 2:**
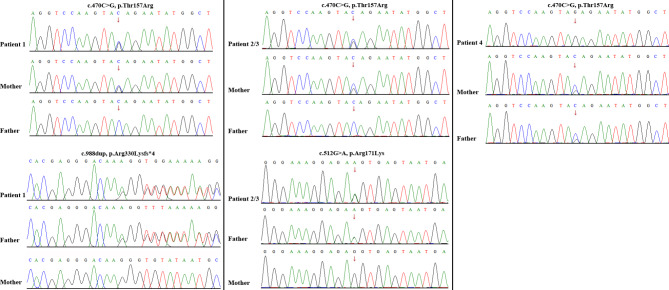
Variant identification by Sanger sequencing. Compound heterozygous *TARS2* variants, c.470 C > G (p.Thr157Arg) (maternally inherited) and c.988dup (p.Arg330Lysfs*4) (paternally inherited), in patient 1 (**a**); c.470 C > G (p.Thr157Arg) (maternally inherited) and c.512G > A (p.Arg171Lys) (paternally inherited), in patient 2 and his sibling (patient 3) (**b**); a homozygous variant, c.470 C > G (p.Thr157Arg) in *TARS2*, in patient 4. Both parents were asymptomatic heterozygous carriers (**c**)

WES identified compound heterozygous variants, c.470 C > G, p.Thr157Arg and c.988dup, p.Arg330Lysfs*4, in *TARS2* in the proband. The maternally inherited variant (c.470 C > G, p.Thr157Arg) has been previously reported in the literatures [10, 11]. The paternally inherited variant (c.988dup, p.Arg330Lysfs*4) is novel, and is predicted to truncate the aminoacylation domain of *TARS2* (Fig. 2a). Both variants are classified as clinically pathogenic according to the ACMG/AMP guidelines [14].

#### Family 2

Patient 2 was the second child of a nonconsanguineous Chinese couple. The pregnancy was normal except for significantly reduced fetal movement. He was born at full term with normal birth measurements: his length was 49 cm, his weight was 3.64 kg and his head circumference was 34 cm. His Apgar scores were 8, 9, and 9. After birth, he was referred to the clinic due to pneumonia and jaundice. At 1 month of age, he displayed axial hypotonia and feeding difficulties. At 2.5 months of age, he was diagnosed with focal epilepsy, with each seizure lasting for approximately one minute and characterized by staring and rigidity of the upper extremities. EEG showed epileptiform discharges with frequent polyspikes and slow waves. The symptoms could not be effectively alleviated by administering antiepileptic drugs. Serum lactate (7.03 mmol/L) and blood NH3 (73 µmol/L, normal range 9–47 µmol/L) levels were elevated. At 4 months of age, he developed limb hypertonia. MRI showed frontal subdural fluid, bilateral hemispheric brain atrophy, multiple hyperintense lesions in the basal ganglia, globus pallidus, thalamus, midbrain and cerebral peduncles, and a thin corpus callosum (Fig. 1B). He had a growth delay at 6 months of age: his height was 63 cm (<-2 SD), his weight was 6.2 kg (<-2 SD), and his head circumference was 40 cm (-3 SD). He died due to severe respiratory failure at 1 year and 10 months of age.

His elder brother (Patient 3) was also born after an uneventful pregnancy, during with markedly reduced fetal movement was observed. His birth measurement parameters were normal: his weight was 2.6 kg, length was 50 cm, and head circumference was 34 cm. He also exhibited clinical features of mitochondrial encephalopathy that were strikingly similar to those of his younger brother. He had never achieved the ability to raise his head alone and exhibited no eye contact. Increased serum lactate level was regularly monitored. He exhibited delayed growth: his height was 73 cm (<-2 SD), his weight was 9 kg (<-2 SD), and his head circumference was 42.5 cm (<-3 SD) at 1 year and 3 months of age. He died due to respiratory problems, lactic acidosis and severe developmental delay at 2 years and 4 months of age.

WES revealed the recurrent missense variant, c.470 C > G, p.Thr157Arg (maternally inherited), and a novel variant, c.512G > A, p.Arg171Lys (paternally inherited), in *TARS2* in the two affected children. The variant c.512G > A, p.Arg171Lys was located in the N2 domain and was highly conserved among different species, and was absent in in the Genome Aggregation Database (Fig. 2b). The variant was confirmed in trans with the previously identified pathogenic variant c.470 C > G, p.Thr157Arg. Thus, the variant was classified as likely pathogenic according to the ACMG/AMP guidelines [14].

#### Family 3

Patient 4 was a 2-year and 2-month-old female born to unrelated parents. She was born at full term after an uneventful pregnancy. She had normal birth measurements: her length was 50 cm, her weight was 3.3 kg, and her head circumference was 33 cm. She was able to raise her head at 5 months of age. However, the patient presented with progressive developmental regression, including unstable head control and axial hypotonia at 7 months of age, and she gradually displayed limb spasticity after eight months of age. She had obvious feeding difficulties. An increased serum lactate level was detected (4.9 mmol/L). Furthermore, plasma amino acid analysis revealed increased free carnitine (70.28 µmol/L, normal range 9-53.7 µmol/L) and hyperammonemia (98 µmol/L, normal range 18–72 µmol/L). At 5 months of age, she displayed infantile spasms, characterized by brief generalized muscle spasm with straight arms extended and bent torso and legs. The events lasted for 15 s–1 min each time, and eight episodes usually occurred every day. EEG revealed hypsarrhythmia, including a large number of irregular high-voltage, sharp spikes/polyspikes/slow waves. The patient’s epilepsy was not controlled even when sodium valproate or a ketogenic diet was applied. The serum lactate concentration was maintained at a constant level according to real-time monitoring. Brain MRI revealed hyperintensity of the bilateral basal ganglia on symmetrical T2-weighted imaging (T2WI) and a thin corpus callosum at 7 months of age (Fig. 1C). On recent physical examination at 2 years and 2 months of age, she exhibited growth delay: her height was 82 cm (-2 SD), her weight was 10 kg (-2 SD), and her head circumference was 43.5 cm (<-3 SD). She was not able to raise her head and had no language or cognitive development.

WES revealed a homozygous variant, c.470 C > G, p.Thr157Arg in *TARS2*, in the patient. Both parents were asymptomatic heterozygous carriers (Fig. 2c).

## Discussion

Biallelic pathogenic variants in *TARS2* are the genetic cause of combined oxidative phosphorylation deficiency 21 (COXPD21). To date, only 28 variants in *TARS2* have been reported in 28 COXPD21 patients from 24 unrelated families [1, 9–13]. Here, we described additional four patients from three unrelated Chinese families who displayed psychomotor delay, axial hypotonia, limb hypertonia, intractable epilepsy, increased serum lactate levels and abnormal brain MRI. Patients were diagnosed with mitochondrial encephalomyopathy by experienced clinical specialists. WES revealed a recurrent missense variant (c.470 C > G, p.Thr157Arg) and two novel variants (c.512G > A, p.Arg171Lys and c.988dup, p.Arg330Lysfs*4). These variants were classified as clinically likely pathogenic/ pathogenic and were related to our patients’ manifestations. These newly identified variants further expand the *TARS2* mutation spectrum and improve the molecular diagnosis of COXPD21.

**Fig. 3:**
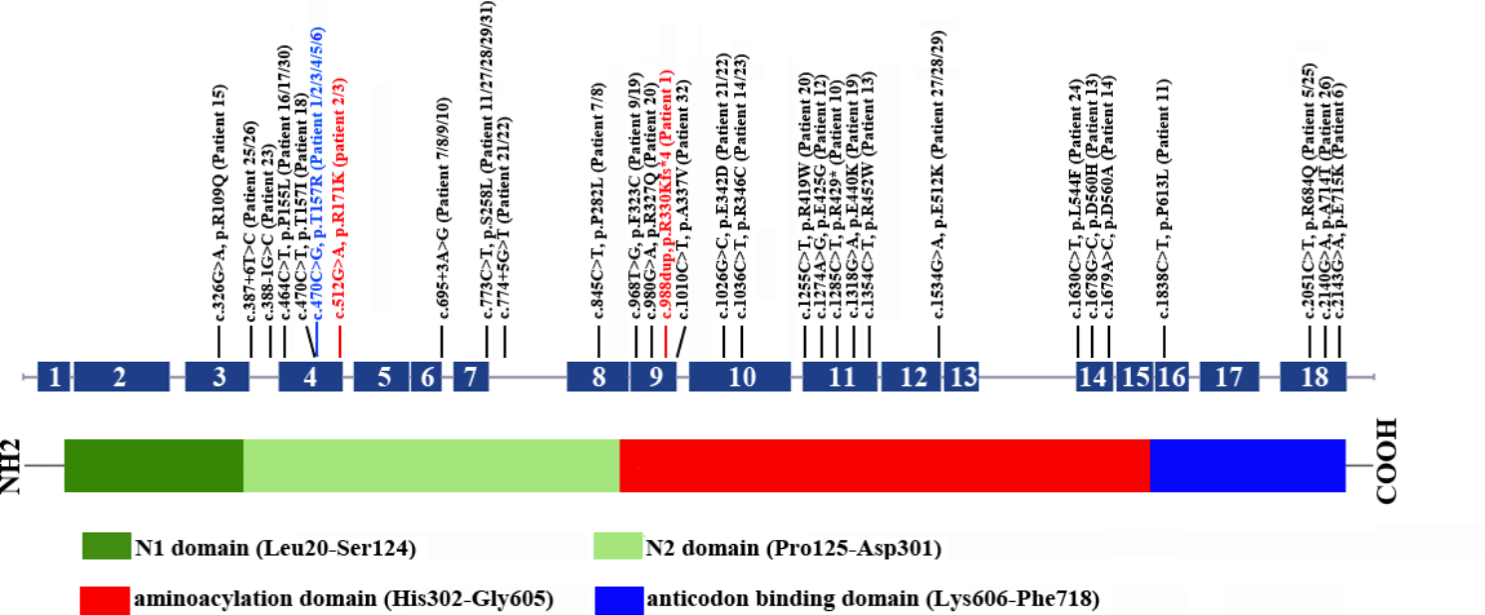
Schematic representation of TARS2 variants identified to date. The structure of TARS2 contained 18 exons (dark blue rectangles), and introns (grey horizontal line); lower side: the TARS2 protein domains: N1 domain; N2 domain; Aminoacylation domain; Anticodon binding domain; motif. The localization of variants and substitutions identified is signed with vertical line. Black: Variants identified in the literature; Red: Novel variants detected in this study. Blue: the founder mutation in Chinese patients with COXPD21

To date, a total of 30 *TARS2* variants, including the two novel variants reported in this study, have been identified [1, 9–13]. *TARS2* null variants (nonsense, frameshift and splicing) and missense variants accounted for 20% and 80%, respectively. These variants are distributed in the N1 domain (3.3%), N2 domain (33.3%), aminoacylation domain (50%) and anticodon binding domain (13.3%) (Fig. 3). Patients carrying *TARS2* null variants (Patients 1, 7, 8, 9, 10), especially biallelic null variants (Patient 10), are more likely to suffer from early-onset severe clinical phenotypes with early death (usually less than one year old). Individuals carrying *TARS2* biallelic missense variants presented with later-onset manifestations with longer survival, except for Patients 2, 3, 15, 18 and 30, who may have been affected by complications that were not promptly corrected [1, 9–13]. Null variants may lead to more severe clinical outcomes than missense variants owing to complete loss of protein function. According to previous reports in Chinese patients, the c.470 C > G, p.Thr157Arg variant recurred in six Chinese patients [10, 11]. According to the Genome Aggregation Database, this variant was detected only in East Asians, with an allele frequency of 0.00001193 (3/251430), but it recurred in affected patients who were either heterozygous or homozygous. This result suggests that c.470 C > G is a Chinese-specific founder mutation with a frequency of 1.8/10^4^ in the Chinese population based on the cumulative allele frequency in one commercial Chinese genetic testing database (*n* = 152,423).

Next, we analyzed the clinical phenotypes of all COXPD21 patients (Supplementary Table 1) [1, 9–13]. The main clinical features included psychomotor development delay (30/31), axial hypotonia (27/29), limb hypertonia (21/27), developmental regression (12/32), absent speech (21/30), epilepsy (17/28), growth delay (14/26), microcephaly (10/24), eye anomalies (10/25), increased serum lactate (21/23), brain MRI anomalies (25/28) and EEG anomalies (11/13). The other phenotypes included hearing loss (4/25), feeding difficulties (9/9), no/poor eye-eye contact (8/8), respiratory problems (6/8), reduced fetal movement (4/4), hyperhidrosis (2/7), sleep irregularities (1/2) and echocardiography anomalies (6/24). Neuropsychiatric anomalies and increased serum lactate levels occur in almost all reported COXPD21 patients. Epilepsy is a common phenotype of COXPD21. All of patients with epilepsy except for Patient 24 suffered from intractable epilepsy with no response to antiepileptic drugs [1, 10, 11, 13]. Developmental regression, absent speech, growth delay, microcephaly, feeding difficulties, no/poor eye-eye contact and respiratory problems were frequently described in COXPD21 patients, which should be added in OMIM phenotypic spectrum of COXPD21 [1, 9–13]. Notably, eye anomalies, including nystagmus and strabismus, have been observed in previously reported patients [1, 13]. Patient 5 underwent further follow-up by Yuan (Haiming Yuan is the corresponding author of the literature) [11]. Now, Patient 5 was 4.5 years old. Brief episodes of tonic upward eye deviation and visual inattention were observed. Three patients (Patients 10, 13 and 27) also exhibited the similar features [1, 13]. It added to the known eye anomaly features of COXPD21 [1]. Reduced fetal movement was previously reported in Patient 5, which was also observed in three patients (Patients 1, 2 and 3) in this study, which further confirmed that reduced fetal movement is one of the characteristics of COXPD21. Furthermore, Patient 5 displayed obvious sleep irregularities beginning at 2 years of age. This phenotype was not described in other patients either because some patients were too young to exhibit the trait or because the phenotype was ignored during clinical evaluation, which deserves further investigation. In summary, the novel phenotypes greatly enriched the clinical features of COXPD21.

## Conclusions

We identified two novel *TARS2* variants in our COXPD21 patients, which expands the genetic variant spectrum of *TARS2*. Our study suggested that *TARS2* c.470 C > G is a Chinese-specific founder mutation and should be considered for inclusion in a Chinese carrier screening panel. Our patients exhibited novel phenotypes, including eye anomalies, reduced fetal movement and sleep irregularities. These findings will deepen our understanding of the clinical characteristics of COXPD21 patients and guide their clinical management and genetic counseling.

## Electronic supplementary material

Below is the link to the electronic supplementary material.

Supplementary Material 1

## Data Availability

The datasets used and analyzed during the current study are available from the corresponding author on reasonable request. The data are not publicly available due to privacy or ethical restrictions.

## Abbreviations

COXPD21: Combined oxidative phosphorylation deficiency 21
ME: Mitochondrial encephalomyopathy
TARS2: Threonyl tRNA-synthetase 2
WES: Whole-exome sequencing
ACMG/AMP: American College of Medical Genetics and Genomics/Association for Molecular Pathology
